# Single stage cricoid split laryngoplasty with costochondral rib grafting is a novel approach to treat subglottic stenosis in a paediatric patient: A case report

**DOI:** 10.1016/j.ijscr.2024.109952

**Published:** 2024-06-27

**Authors:** Khalid Maudood Siddiqui, Umair Aftab Baig, Muhammad Saad Yousuf

**Affiliations:** Department of Anaesthesiology, Aga Khan University, Karachi, Sindh, Pakistan

**Keywords:** Cricoid split laryngoplasty, Costochondral rib grafting, Paediatric anaesthesia subglottic stenosis, Stridor in children, Single stage cricoid split laryngoplasty

## Abstract

**Introduction and importance:**

Subglottic stenosis (SGS) appears to be a commonly encountered condition in the paediatric age group. Single stage cricoid split laryngoplasty with costochondral rib grafting in paediatric patients is a unique, innovative, and advanced operation in nature. Morbidity and mortality rates can be minimized with early diagnosis and prompt treatment.

**Presentation of case:**

Presenting the case of a 13-month-old child diagnosed with Grade II SGS who was managed for cricoid split laryngoplasty with a costochondral rib graft. It was a unique strategy for providing infants and neonates with symptomatic SGS with a safe and efficient substitute for long-term tracheostomy. When healing was completed, the patient regained the function of their airway. The approach was successful, and preventable to long-term tracheostomy.

**Discussion:**

Performing this procedure early in children has shown higher rates of success and it is safe and effective. Further extensive research and studies need to be conducted in this domain, and every patient's status should be reviewed time and again to tend to their specific needs, and the choice of procedure should be made optimally based on clinical evaluations.

**Conclusion:**

Successful management of a 13-month-old child with Grade II subglottic stenosis through cricoid split laryngoplasty with costochondral rib grafting is a challenging and novel approach to treating single-stage SGS.

## Highlights

• Laryngoplasty in single-stage surgery particularly in paediatric patients is unique in nature.
• It is an innovative and advanced operation comprising an anterior/posterior cricoid split accompanied by a cartilage graft.
• Performing this procedure earliest possible has shown higher rates of success and it is safe and effective.

## 1 Introduction

Subglottic stenosis (SGS) is defined as a narrowing of the lumen of the larynx in the subglottic region, commonly extending to the glottis [1]. It is a condition of the paediatric age group, involving particularly the neonates [2,3]. It commonly obstructs the air passages and increases the work of breathing and is broadly divided into two categories; congenital (1 in 4000 live births) and acquired (2 %–8 %). Clinically presenting as variable dyspnea depending upon the severity of the stenosis [4,5].

Definitive treatment is centered on surgical intervention. Paediatric airway surgery encompasses a broad utilization of endoscopic and split-open procedures that can be used differently in managing various grades of SGS. The pre-requisite for operative procedures is patient's airway management during anaesthesia which comes with its risks and complications.

## 2 Presentation of case

A 13-month-old child with a weight of 10 kg was admitted to the emergency room (ER) of the hospital with a history of nasal blockage for 15 days, accompanied by complaints of chest congestion, croupy voice, stridor in the past week, and shortness of breath for one day. The parents stated that five days prior, the child had also chewed nuts.

Initially, foreign body aspiration and croup were kept in the differential diagnosis; foreign body aspiration was excluded after clinical workup; the child was managed as a croup and was discharged the next day. The patient had a second episode of stridor and croupy voice a day ago and was brought again to the hospital. The treatment included epinephrine nebulization with dexamethasone, and the patient was relieved and sent home. The patient was presented a third time in a lethargic condition to the ER with complaints of croupy voice, respiratory distress, and central cyanosis with pallor since morning.

Clinical examination revealed a severe respiratory stridor with bilateral wheezes and decreased air entry on the left side. Nebulization with epinephrine and cold saline was given, along with an intramuscular dexamethasone injection. Oxygen saturation was 80 %, on that occasion, the patient was rushed to the operating room (OR), hurriedly intubated, and shifted to the surgical intensive care unit (SICU) for further management.

**Fig. 1:**
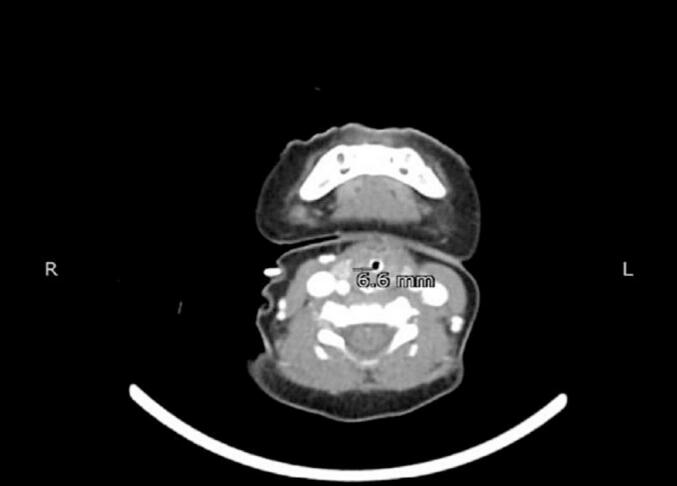
CT scan of neck (axial view).

The patient was kept sedated in the SICU. The presence of a foreign body was excluded after evaluation by the paediatric surgeon. CT scan of the neck and chest with contrast was done which revealed a well-defined circumferential thickening of the airway, extending from the level of the hyoid bone inferior to the level of the thyroid gland, involving the supra glottis and glottis of the larynx, likely due to edema (Fig. 1).

The patient was weaned off from the ventilator after two days and extubation was done, but after a few hours, the patient was reintubated again in the OR due to respiratory distress. While in the OR, video laryngoscopy and bronchoscopy were also done, and the findings revealed a Grade II Subglottic Stenosis. The patient was reintubated again with a 3.5 mm cuffed endotracheal tube (ETT) and shifted to the SICU. The ENT surgeon was taken on board and considered different options, like balloon dilatation, useful for grade I and grade II subglottic stenosis, unless there is a fixed cartilaginous obstruction in which an open approach is recommended. This patient had grade II subglottic stenosis with a fixed cartilaginous obstruction; therefore, an open approach was selected. Standard tracheostomy and T-tube insertion options were refused by parents, despite many sessions of counselling. Finally, “Cricoid Split Laryngoplasty with Costo-chondral Rib Grafting,” was planned under general anaesthesia and the patient was kept nothing per oral (NPO).

As anaesthetic management, the American Society of Anaesthesiologists (ASA) status was assessed and labelled ASA III E. Respiratory system evaluation revealed peri-hilar vascular congestion, while the rest of the systemic evaluation was normal. The patient was shifted to the OR for a planned procedure. Standard II monitoring was applied including ECG, NIBP, SpO_2_, and ETCO_2_. Intravenous (IV) propofol 1.5 mg/kg and cisatracurium 0.2 mg/kg were given for induction and muscle relaxation, from an already placed 24 G cannula in the left hand. The laryngoscopy was done with a video laryngoscope, and a 3.5 mm cuffed endotracheal tube (ETT) was inserted in the trachea. IV fentanyl 2 μgm/kg was also given for analgesia. Anaesthesia was maintained with oxygen 40 %, nitrous oxide 60 % and isoflurane 1–2 %. An intermittent apnea technique was used to facilitate the surgery.

**Fig. 2:**
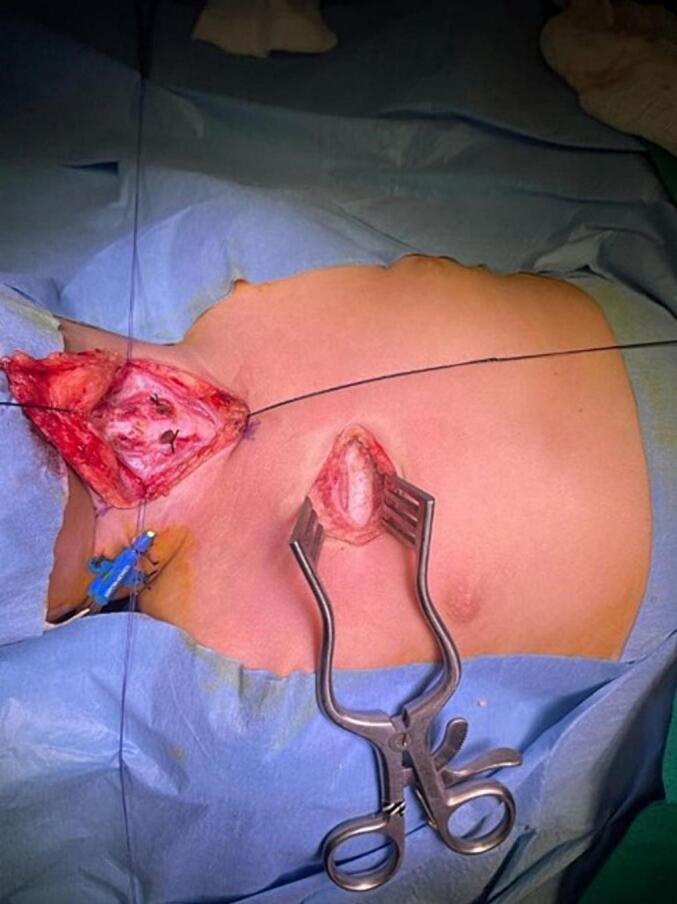
Cricoid split laryngoplasty with costochondral rib grafting.

As a surgical procedure, incisional laryngofissure was made to cut and open the thyroid cartilage along with the cricoid cartilage at its lower end. The circumferential diameter was widened by the insertion of a costochondral graft, the cartilage which was taken from the second rib (Fig. 2). This was followed by the replacement and fixation of the nasotracheal tube of size 4 mm from 3.5 mm.

Postoperatively the patient was kept sedated and relaxed on mechanical ventilation for five days to avoid postoperative complications that occurred after cricoid split laryngoplasty. After five days the patient was weaned from mechanical ventilation and extubated successfully. After extubation, the patient was breathing normally without dyspnea and respiratory distress.

## 3 Discussion

Otolaryngologists face difficulties in managing SGS, particularly in the paediatric population. These are frequently complicated cases that require multiple methods of therapy to fully cure. A few factors need to be taken into account, such as the patient's neurological condition, nature, location, and severity of the stenosis, the level of airway obstruction, and the existence of vocal cord damage [6].

We have presented a specifically challenging case regarding anaesthesia management. The patient's response to being extubated and reintubated for surgery manifested in a derangement of the patient's critical condition. Anaesthesia administration is a particularly hard task owing to the already narrowed airway, which calls for great expertise, close observation, and alteration of the parameters as and when deemed essential to meet the special requirements of a patient.

Surgical intervention is risky because of the age group involved and must be chosen appropriately to best suit the needs of the patient and yield promising results with the least chance of diving into a handful of complications. There are various procedures available for laryngotracheal reconstruction (LTR), partial cricotracheal resection (PCTR), and extended cricotracheal resection (ECTR). All these interventions are highly invasive and remove a significant amount of tissue from the larynx and/or trachea, which calls for the need for replacement by stenting or grafting [7]. This case was planned for “Cervical Tracheoplasty” but changed in favor of “Cricoid Split Laryngoplasty” involving costochondral rib grafting. The most effective method for treating paediatric patients with advanced grade (grade III or grade IV) subglottic stenosis remains laryngotracheoplasty with cartilage graft [8]. The outcome was promising, and no postoperative debilitating sequelae were noted. The patient showed resolution of the symptoms of respiratory compromise, was successfully extubated and breathed normally.

We have performed an innovative and advanced laryngoplasty operation, which includes an anterior or posterior cricoid split and the placement of a cartilage graft. Performing this procedure early in children has shown higher rates of success and it is safe and effective. Single stage cricoid split laryngoplasty with costochondral rib grafting in paediatric age group demonstrates the appropriateness and avoids tracheostomy showing the novelty and strength of the procedure. This case is in the present literature and is an example of effective dealing with subglottic stenosis paediatric population developments in airway management away from intubation and positive pressure airway support. The success of this procedure has been hailed by most literature and has also become evident by this case report. There have been reports of the collapse of the cartilage grafts. Still, further extensive research studies need to be conducted in this domain, and every patient's status should be reviewed time and again to tend to their specific needs, and the choice of procedure should be made optimally based on all these evaluations [9].

## 4 Conclusion

Successful management of a 13-month-old child with Grade II subglottic stenosis through cricoid split laryngoplasty with costochondral rib grafting is a challenging and novel approach to treating single-stage SGS. Anaesthetic workup and the procedure account for the most important components in the management paradigm and explore innovative approaches specifically tailored to fit the patient's needs.

This study has been reported according to the SCARE criteria [10].

## Parental consent for minors

Written informed consent was obtained from the patient's parents/legal guardian for publication and any accompanying images. A copy of the written consent is available for review by the Editor-in-Chief of this journal on request.

## Funding

This research did not receive any specific grant from funding agencies in the public, commercial, or not-for-profit sectors.

## Ethical approval

The study was exempted from ethical approval by the Aga Khan University.

## Author contribution

Study conceptualization and methodology: Khalid Maudood Siddiqui.

Data collection: Umair Aftab Baig.

Data analysis and interpretation: Muhammad Saad Yousuf.

Writing the paper: Umair Aftab Baig.

## Guarantor

Khalid Maudood Siddiqui.

## Conflict of interest statement

None.
